# Enlarging purpuric plaques on an elderly patient

**DOI:** 10.1016/j.jdcr.2024.11.039

**Published:** 2024-12-24

**Authors:** Kristen Fernandez, Robin Wang, David Eilers

**Affiliations:** a Stritch School of Medicine, Loyola University, Maywood, Illinois; b Division of Dermatology, Loyola University Medical Center, Maywood, Illinois; c Section of Dermatology, Edward Hines, Jr Veterans Affairs Hospital, Hines, Illinois

**Keywords:** chronic lymphocytic leukemia, dermoscopy, leukemia cutis, linear-irregular vessels, polymorphous vessels, serpentine vessels

## Case description

**Fig 1:**
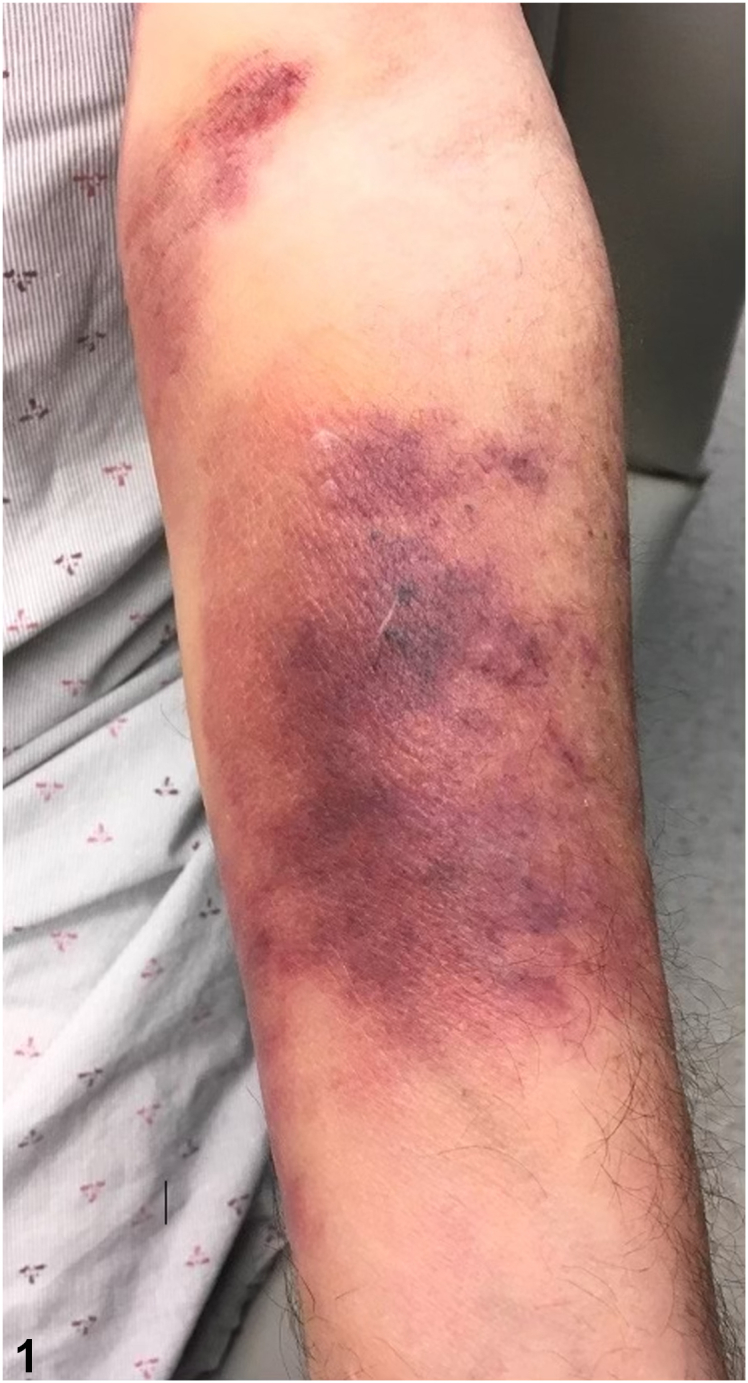

**Fig 2:**
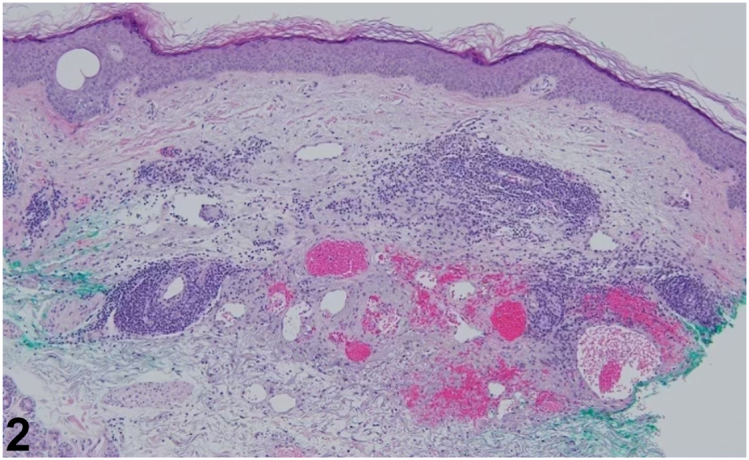

A 76-year-old man with chronic lymphocytic leukemia, not undergoing active treatment, presented with a 1-month history of a painless, enlarging purpuric eruption on the left forearm. The patient denied history of trauma, prior treatment to the area, or new systemic symptoms. Physical examination revealed violaceous and telangiectatic purpuric plaques (Fig 1) and an enlarged left cervical lymph node. Histology demonstrated foci of small hyperchromatic lymphocytes expressing CD20, CD5, and CD23 in a background of solar elastosis (Fig 2). HHV-8 staining was negative.

**Question 1: What is the diagnosis?**

A. Cutaneous T-cell lymphoma
B. Kaposi sarcoma
C. Arteriovenous malformation
D. Vasculitis
E. Leukemia cutis

**Answers:**

A. Cutaneous T-cell lymphoma – Incorrect. While both leukemia cutis and cutaneous T-cell lymphoma involve lymphocyte infiltration, they typically present differently. Cutaneous T-cell lymphoma often manifests as patches, plaques, or tumors that are usually associated with erythema and scaling. Additionally, the immunophenotype differs, as cutaneous T-cell lymphoma primarily expresses T-cell markers, not B-cell markers seen in this case.
B. Kaposi sarcoma – Incorrect. Kaposi sarcoma typically presents with violaceous macules and nodules, often associated with HHV-8 infection, especially in immunocompromised patients. However, in this case, HHV-8 staining was negative, and the clinical presentation aligns more with leukemia cutis.
C. Arteriovenous malformation – Incorrect. Arteriovenous malformations are abnormal connections between arteries and veins that can present as vascular lesions on the skin. While they can appear as purpuric or bluish lesions, Arteriovenous malformations typically have distinct features such as pulsatility or a bruit, and they do not involve the neoplastic lymphocyte infiltration characteristic of leukemia cutis.
D. Vasculitis – Incorrect. Vasculitis is an inflammatory condition affecting the blood vessels and often presents with systemic symptoms, such as fever, malaise, or joint pain, along with skin findings. Common clinical signs include erythematous macules, papules, and purpura, often evolving into ulcerated or necrotic lesions. In more severe cases, necrosis or ulceration may occur, particularly in areas with poor blood supply.
E. Leukemia cutis – Correct. This condition is characterized by the infiltration of the skin by neoplastic leukocytes, most commonly seen in patients with hematologic malignancies such as chronic lymphocytic leukemia.^1^ In this case, the patient's history of chronic lymphocytic leukemia and the presence of violaceous purpuric plaques are consistent with leukemia cutis. The histologic findings of hyperchromatic lymphocytes expressing CD20, CD5, and CD23 further support this diagnosis.

**Question 2: What dermoscopic findings would be most characteristic of leukemia cutis in this patient?**

A. Diffuse erythema without vascular structures
B. Nonhomogenous arborizing vessels
C. Scattered dotted vessels
D. Polymorphous vascular pattern with linear braching vessels
E. Hairpin vessels with white halos

**Answers:**

**Fig 3:**
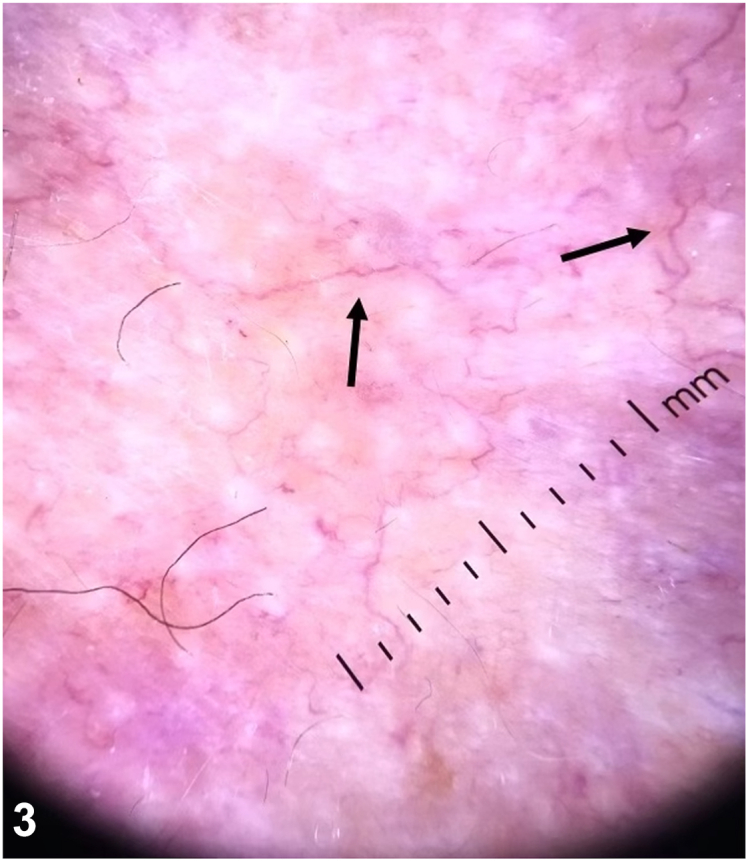
E. Hairpin vessels with white halos – Incorrect. Hairpin vessels surrounded by white halos are commonly observed in keratinizing tumors such as squamous cell carcinoma, seborrheic keratosis, and keratoacanthoma.^3^ These vessels appear as looped structures and are not characteristic of leukemia cutis.

A. Diffuse erythema without vascular structures – Incorrect. Diffuse erythema without distinct vascular structures is nonspecific and can be seen in various inflammatory conditions. Previous research has discussed the limitations of using erythema alone as a diagnostic feature and the need for specific vascular patterns to diagnose leukemia cutis accurately.^2^
B. Nonhomogenous arborizing vessels – Incorrect. Arborizing vessels are typically seen in basal cell carcinoma.^3^ They represent a more organized pattern of vascularization that does not indicate leukemic infiltration. Arborizing vessels are often associated with specific types of basal cell carcinoma, thereby differentiating them from the chaotic vascular patterns seen in leukemia cutis.
C. Scattered dotted vessels – Incorrect. Scattered dotted vessels are not specific to leukemia cutis. They can be observed in various conditions, including melanocytic lesions and various small, vertically oriented, keratinized lesions, including verruca vulgaris, actinic keratosis, seborrheic keratosis, Bowen's disease, and squamous cell carcinoma.^3^
D. Polymorphous vascular pattern with linear branching vessels – Correct. This patient’s dermoscopic examination revealed a polymorphous vascular pattern, including linear vessels with branches (Fig 3). There are limited data in the literature about the vascular pattern of leukemia cutis. There have been 7 patient cases that have described dermoscopic findings in adult LC: the majority of cases showed the presence of polymorphous vessels, as demonstrated in our case.^4^

**Question 3: What are the typical treatment options for leukemia cutis?**

A. Topical corticosteroids
B. Systemic chemotherapy for underlying leukemia
C. Radiation therapy
D. Surgical excision
E. Phototherapy

**Answers:**

A. Topical corticosteroids – Incorrect. While topical corticosteroids may reduce inflammation and manage localized skin symptoms, they do not address the underlying neoplastic process. Therefore, they are not considered a primary treatment for leukemia cutis. Their use might be supportive but is insufficient for managing the condition.
B. Systemic chemotherapy for underlying leukemia – Correct. The primary treatment for leukemia cutis focuses on addressing the underlying hematologic malignancy. Systemic chemotherapy is often utilized to target the leukemia itself, which can lead to resolution of the cutaneous manifestations. Treatment regimens may vary based on the type and stage of leukemia.^1^^,^^2^
C. Radiation therapy – Incorrect. Radiation therapy is not a standard treatment for leukemia cutis. It may be used in specific situations, such as localized skin involvement or palliative care for symptomatic relief, but it is not typically employed as a first-line treatment for leukemia cutis.^2^
D. Surgical excision – Incorrect. Surgical excision is generally not indicated for leukemia cutis, as the lesions are a manifestation of systemic disease rather than localized tumors. Surgical intervention may be reserved for specific cases where a cutaneous lesion needs to be removed for diagnostic or cosmetic reasons but does not address the underlying leukemia.
E. Phototherapy – Incorrect. While ultraviolet light B can be useful in local control of the skin lesions,^2^ this therapy is mainly used for inflammatory skin conditions such as psoriasis or eczema and is not a standard treatment for leukemia cutis. It does not target the underlying neoplastic process and is therefore not appropriate in this context.

## Conflicts of interest

None disclosed.

## References

[1] Parsi M., Go M.S., Ahmed A. (2024). StatPearls [Internet].

[2] Robak E., Braun M., Robak T. (2023). Leukemia cutis-the current view on pathogenesis, diagnosis, and treatment. Cancers.

[3] Ayhan E., Ucmak D., Akkurt Z. (2015). Vascular structures in dermoscopy. An Bras Dermatol.

[4] Sławińska M., Sokołowska-Wojdyło M., Biernat W., Zaryczańska A., Nowicki R.J., Sobjanek M. (2021). Dermoscopic features of leukemia cutis-case series. Indian J Dermatol.

